# Social Support and Modelling in Relation to Physical Activity Participation and Outdoor Play in Preschool Children

**DOI:** 10.3390/children6100115

**Published:** 2019-10-17

**Authors:** Anne Kerstin Reimers, Karolina Boxberger, Steffen C. E. Schmidt, Claudia Niessner, Yolanda Demetriou, Isabel Marzi, Alexander Woll

**Affiliations:** 1 Department of Sport Science and Sport, Friedrich-Alexander-University Erlangen-Nuremberg, Gebbertstrasse 123b, 91058 Erlangen, Germany; isabel.marzi@fau.de; 2 Institute of Human Movement Science and Health, Faculty of Behavioral and Social Sciences, Chemnitz University of Technology, Straße der Nationen 62, 09111 Chemnitz, Germany; 3 Institute of Sports and Sports Science (IfSS), Karlsruhe Institute of Technology, 76131 Karlsruhe, Germany; steffen.schmidt@kit.edu (S.C.E.S.); claudia.niessner@kit.edu (C.N.); alexander.woll@kit.edu (A.W.); 4 Department of Sport and Health Sciences, Technical University of Munich, 80992 Munich, Germany; yolanda.demetriou@tum.de

**Keywords:** exercise, child, parents, social environment, Germany, child care

## Abstract

Physical activity during early childhood is a prerequisite for healthy development in many cases. The aim of this study was to assess the relationships of social modelling and support from parents, peers, and siblings and domain-specific physical activity participation in a nationwide sample of preschool boys and girls from Germany. 519 preschool children aged 4–6 and one of their parents participated in the ‘MoMo’ Wave 1 Study between 2009 and 2012. Participants and their parents provided self-reported data on social support modelling, and domain-specific physical activity participation (physical activity in sports clubs, physical activity outside of sports clubs, and outdoor play). Parental, peer, and sibling support and modelling were related to domain-specific physical activity: Parental support was particularly relevant for physical activity in sports clubs, and peer support for outdoor play. Parental modelling was only related to physical activity outside of sports clubs: Maternal modelling was a positive correlate in girls and paternal modelling in boys, respectively. Sibling and peer modelling were especially relevant for physical activity in sports clubs. The results were heterogeneous regarding types and providers of support and modelling. Thus, different providers and types of support should be targeted in physical activity promotion programs for preschool children.

## 1. Introduction

Physical activity and outdoor play are essential in early childhood and contribute to a healthy development, as they enable cognitive, physical, emotional, social, and motor learning, as well as well-being [1,2]. Especially in early childhood, extensive physical activity and bodily movement are necessary to develop a sound base for lifelong health and active living throughout one’s lifespan [3]. Thus, the German National Guidelines on Physical Activity recommend at least 180 minutes of physical activity per day in children aged 4–6 years [4].

However, 2 out of 3 preschool children in Germany [5,6] are not as physically active as recommended by the guidelines, with a higher risk of inactivity in girls compared to boys [5,7,8]. A large accelerometer study with over 700 participants conducted in a federal state in Germany, Baden-Württemberg, revealed that preschool children spend 32.55 and 20.76 min per weekend day and weekday afternoon in moderate-to-vigorous physical activity (MVPA), respectively [6]. Nevertheless, in Germany in the preschool age group (age 4–6), the proportions of children who are regularly physically activity is still higher compared to older children and adolescents and physical activity levels decline with advancing age [5]. Current longitudinal results indicate that the overall amount of physical activity, as well as MVPA in children already declines rapidly from age seven onwards [9]. Thus, the promotion of physical activity and the creation of opportunities for supporting the natural desire for movement should start at an early stage in life [10].

The family environment plays an important role for providing opportunities for physical activity and for providing a less restrictive environment in which children can act out their urge to move [11,12,13]. Although young children spend a large amount of their day in preschools or child care settings, during their leisure time they are reliant on support of their parents and their social environment when they want to participate in physical activities or do sports [14]. Especially, playing outside requires social support (e.g., supervision of parents), as a number of young children are mostly not permitted to go outside on their own without adult supervision [15,16]. Additionally, parental restrictions may hinder physical activity participation and outdoor play in preschool children [11].

As proposed by the Social Cognitive Theory [17], learning occurs in a social context with a dynamic and reciprocal interaction of the individual, behavior and environment. Individuals acquire and maintain behavior in a unique way, while also considering the social environment in which individual perform the behavior. Therefore, in the development of behavior patterns, young children tend to imitate the social behavior of their social models, who are significant persons (e.g., parents, peers or siblings) exemplifying daily behavior routines like leisure-time activities. Same-sex imitation hypothesis indicates that children prefer to imitate behaviors of same-sex models or their same-sex parent, respectively [18,19]. Besides social modelling, social support is presumed to be a relevant social environmental facilitator of childhood behavior: Following the theoretical basis from Uchino [20], four functions of social support encompassing emotional, instrumental/tangible, informational, and companionship support are related to physical activity. In general, social support and social modelling have been shown to be consistent predictors of physical activity in people of different age groups [21]. Especially, young people rely on a supportive social environment concerning their opportunities to be physically active [22].

However, despite the need to promote physical activity and prevent sedentary behaviors from early childhood on, previous research has sparsely focused on the relationships of social environmental factors on physical activity behaviors in preschool children. Furthermore, previous studies investigating the effects of the social environment have mainly concentrated on overall or leisure-time physical activity [23,24], but did not take the different domains of physical activity and play behavior into account [25], although the relationships of social environmental factors with physical activity may depend on the domain of physical activity as shown in adolescents in a previous study [26]. Regarding Germany, preschool children participate in physical activities in different domains: Nearly 50% of children aged 4–5 years are members in sport clubs [8]. In addition, beside physical activity in sports clubs, 42% of boys and 39% of girls in Germany engage in unorganized sport activities such as playing outside. Thus, research should distinguish between correlates of physical activity in the different domains.

The aim of the present study was to analyze social environmental correlates of physical activity participation and outdoor play in a nationwide sample of preschool children from Germany by taking different domains of physical activity into account. Based on previous findings we hypothesized that (1) social modelling and (2) social support of parents, peers, and siblings are related to physical activity participation in different domains in preschool-age boys and girls.

## 2. Materials and Methods

### 2.1. Study Design

The data for this study was obtained from a subsample of the German nationwide MoMo-Study on physical fitness and physical activity in children and adolescents from Germany, and its umbrella study, the German Health Interview and Examination Survey for Children and Adolescents, KiGGS [27,28]. For both studies, a nationwide diverse sample of children and adolescents from Germany was planned. Thus, a stratified, multi-stage sample with three evaluation levels was drawn [29]. First, a systematic sample of 167 primary sampling units was selected from an inventory of German communities stratified according to the BIK classification system that measures the level of urbanization and geographic distribution [28]. Second, an age-stratified sample of randomly selected children and adolescents was drawn from the official registers of local residents. A total of 12,368 children and adolescents participated in the KiGGS Wave 1 [30], of which 6,076 were randomly assigned to MoMo Wave 1. Thus, the MoMo Study is based on a subsample of the KiGGS Study. The final number of preschool children aged 4–6 years in MoMo Wave 1 was 519, building the final sample of this study. Response rates of the MoMo Study are reported elsewhere [31].

### 2.2. Data Collection

MoMo Wave 1 data was collected between 2009 and 2012. The data collection took place over three years, because a large representative nationwide sample was drawn for KiGGS and the MoMo Study [27,28]. The data collection teams had to go to the 167 study sides, which were distributed over the whole of Germany. In order to avoid systematic bias in the study results by regional or seasonal trends, the sequence of sample points visited for data collection was laid down beforehand in a random route planning.

Parents and their children were invited to examination rooms at central locations in the 167 cities and municipalities that were within close proximity of their homes. A parent gave written informed consent and then completed questionnaires on physical activity behaviors, social support, social modelling, and socio-demographic aspects together with the child in the presence of a qualified interviewer on site. The KiGGS and the MoMo Studies were approved by the Charité/Universitätsmedizin Berlin ethics committee and the Federal Office for the Protection of Data and were conducted according to the Declaration of Helsinki [32].

### 2.3. Measures

The measures on physical activity participation and the social environment were captured within the MoMo Study, while confounding factors (socio-demographic variables) were captured within the KiGGS Study.

#### 2.3.1. Physical Activity Participation

We used the MoMo Physical Activity Questionnaire (MoMo-PAQ) preschool version to assess habitual physical activity in different domains (physical activity in sports clubs, leisure-time physical activity outside of sports clubs, and outdoor play) in preschool children [33]. The MoMo-PAQ consists of 28 items and measures different domains of physical activities in a habitual week. Data obtained with the MoMo-PAQ are sufficiently valid and reliable (test-retest reliability: *ICC* = 0.68). Further information on the MoMo-PAQ is reported elsewhere [33,34].

Participants were asked if they regularly participate in physical activity in sports clubs. They could list up to four different physical activities in sports clubs they regularly engage in. A dichotomous variable “physical activity in sports clubs” was built according to 1—“regular participation in physical activities in sport clubs” or 0—“no physical activities in sport clubs”.

Additionally, participants were asked to if they regularly participate in physical activities outside of sports clubs (e.g., swimming in a public pool with parents, Inline skating, or cycling). They could state up to four unorganized, leisure-time physical activities taking part outside of sports clubs. A dichotomous variable “physical activity outside of sports clubs” was built according to 1—“regular participation in physical activities outside of sports clubs” or 0—“no physical activities outside of sports clubs”.

We used an 8-scaled item about days per week in which the child plays outside (“How often do you normally play outside during a week (for example: playing tag, skipping rope, or going to the swimming pool)?”) and an item about minutes spent on average during one of those days to assess unorganized outdoor play. According to the WHO guidelines on physical activity [35], a dichotomous variable “daily outdoor play” was built according to 1—“seven days per week with outdoor play” or 0—“no to six days per week with outdoor play”

#### 2.3.2. Parental, Peer and Sibling Modelling

Paternal, maternal and sibling modelling were all measured using a single item (e.g., “Does your father regularly do sports?”), which had a dichotomous answer format of 1—“yes“ and 0—“no“. We also measured peer modelling with a single item (“How many of your friends regularly do sports?“) with a four-point rating scale ranging from 1—“none” to 4—“most of my friends”.

#### 2.3.3. Parental and Peer Support

Parental support scales followed the theoretical basis from Uchino [20]. Each scale included two items which were based on a four-point rating scale (e.g., for emotional support: “How important is sport in your family?” 1—“not at all” to 4—“very important”). We assessed peer support by a scale containing three items which were also based on a four-point rating scale (e.g., “How often do your friends ask you if you want to play outside or do sports with them (e.g., playing soccer, riding a bicycle, inline skating)?” 1—“never” to 4—“always”). The scales on social support had good or moderate test-retest reliability over a period of one week. Further information on these measures have been published elsewhere [36].

#### 2.3.4. Confounding Factors

A migration background was assumed if the participant themselves had immigrated to Germany, at least one parent was not born in Germany, or if both parents immigrated to Germany or had no German nationality [37]. Individual-level socioeconomic status (SES) was derived separately for both parents and included items on educational and professional status and the total household income [38]. Children with separated parents were assigned the socioeconomic status of the parent they lived with. Income, educational, and professional status were scored on a scale from 1 to 7, and a sum score was created (range: 3–21) and categorized into low (3–8), medium (9–14) and high (15–21) socioeconomic status [39]. The type of residential area was defined according to the number of residents living in the participants hometown, differentiated into rural area (<5000 residents), small town (5000–19,999 residents), medium-sized town (20,000–99,999 residents), and city (>99,999 residents). Additionally, we captured the “region in Germany”, meaning former east and former west Germany. Furthermore, we also took siblings (yes/no) and the family situation (living at bodily parents/mother/other) into account.

### 2.4. Statistical Analysis

We conducted all analyses with SPSS Version 25. Socio-demographic characteristics were analyzed using descriptive statistics (mean and standard deviation for continuous variables and frequency in percentage for categorical variables). We used chi2-tests and *t*-tests to determine gender differences in social support, social modelling, and physical activity outcome variables.

To analyze the different effects of parental and peer modelling and support on domain-specific physical activity participation stratified by gender, multiple separate logistic regressions were run with the different dichotomous physical activity variables as outcomes and one of the modelling and support variables as a potential correlate. In addition, age, socioeconomic status, region in Germany, residential area, migration background, siblings, and family situation were included as possible confounders in every model. From these logistic regressions, we obtained odds ratios which express the influence of different modelling and support scales on whether a participant is active or not. Higher values express a higher chance of being active in the specific domain with higher amounts of social support or positive modelling. Finally, we ran an additional overall model with every peer and parental modelling and support variable and confounders to determine the overall explained variance for each physical activity outcome variable (R² in Tables 3 and 4). For all analysis, the significance level was set to *p* < 0.05.

## 3. Results

### 3.1. Sample Description

Data from 519 preschool children (244 girls and 275 boys) from the MoMo Study were eligible for analysis in the current study. Socio-demographic characteristics of the participating children are presented in Table 1. The mean age of the preschool children participating in the study was 5.40 (SD = 0.75) years, ranging from 4–6 years. The study population was comprised of 490 (94.4%) native German children and 29 children with a migration background. About 6.2% of the children were assigned to a low, 61.9% to a middle, and 31.9% a high socio-economic position. Most children lived with their biological parents (87.7%).

### 3.2. Social Support and Social Modelling in Relation to Gender

Physical activity, social support, and social modelling variables in boys and girls are presented in Table 2. In the current study, more than half of the preschool children regularly participated in physical activity in sports clubs (54.1%) and in outdoor play activities every day (60.7%). Additionally, 38.0% of the children regularly engaged in physical activity outside of sports clubs. Girls were more likely to participate in physical activity in sports clubs than boys (χ^2^ = 4.406, *p* = 0.036), whereas no significant gender differences were found in physical activity outside of sports clubs and outdoor play.

Regarding gender differences in social environmental variables, girls were more likely to get emotional support from their parents than boys. The highest support scores were found in parental informational support. 67.8% of the children had siblings who were regularly active, half of the children had regularly active mothers, and 42.6% had regularly active fathers.

### 3.3. Social Support and Social Modelling in Relation to Domain-Specific Physical Activity

The results of the logistic regression analyses are presented separately for boys and girls in Table 3 and Table 4, respectively. Results were similar for boys and girls concerning physical activity in sports clubs: parental emotional, informational, and instrumental support and peer and sibling modelling were related to the outcome in both genders. Additional parental companionship support was only related to the outcome in boys (*β* = 2.06, *p* < 0.01).

Regarding the prediction of the other outcome variables in boys, parental emotional (*β* = 2.73, *p* < 0.001), informational (*β* = 2.93, *p* < 0.001), and companionship support (*β* = 3.48, *p* <0.001), as well as peer (*β* = 2.10, *p* < 0.001) and paternal modelling (*β* = 2.17, *p* <0.05) were associated with physical activity outside of sports clubs. Outdoor play in boys was predicted by peer support (*β* = 2.26, *p* <0.001) and parental emotional (*β* = 1.98, *p* < 0.01) and instrumental support (*β* = 1.36, *p* < 0.05), as well as sibling modelling (*β* = 3.59, *p* < 0.001).

In girls, physical activity outside of sports clubs was only predicted by parental companionship support (*β* = 2.11, *p* < 0.01) and maternal modelling (*β* = 2.14, *p* < 0.05), whereas outdoor play was predicted by peer (*β* = 3.08, *p* < 0.001), parental emotional (*β* = 2.01, *p* < 0.05), informational (*β* = 2.38, *p* < 0.01), and companionship support (*β* = 2.65, *p* < 0.01). The explained variance was highest in the physical activity in sport clubs’ models for both boys and girls (R^2^ = 0.597 in boys and R^2^ = 0.465 in girls, respectively).

## 4. Discussion

Using data from the MoMo Study, our present study aimed to identify social environmental correlates (social support and social modelling) of participation in physical activity in and outside of sports clubs and outdoor play. Overall, our results revealed that parental and peer support and modelling, as well as sibling modelling are related to preschool children’s physical activity participation in different domains. Furthermore, we examined differences between boys and girls. However, the results are heterogeneous regarding types and providers of support and modelling behavior. It seems like parental support and modelling of peers and siblings were most important for supporting physical activity in sports clubs in girls and boys. As physical activity in sports clubs was the only domain of structured physical activity, this may indicate that preschool children are particularly in need of support to enable the participation in structured physical activity. Particularly parental instrumental support (e.g., driving children to sports facilities), which has been a strong predictor of physical activity in sports clubs in boys and girls, could be a necessary prerequisite. In contrast, social instrumental support might be less important to make unstructured activities possible, but could encourage them.

Regarding parental support, our results are in line with results published earlier: Loprinzi and Trost [40] found a positive relationship between parental support and preschool children’s home physical activity, but not for MVPA in child care. Schoeppe and Trost [41] differentiated between maternal and paternal support for preschool children’s physical activity and found that both maternal and paternal support were correlates of preschool children’s physical activity, whereby stronger associations were found in girls in comparison to boys. However, they did not differentiate between different domains.

In the present study, parental modelling was only related to physical activity outside of sports clubs. Precisely, girls’ physical activity outside of sports clubs was associated with maternal modelling and boys’ with paternal modelling, which corroborates the same-sex hypotheses [18]. Other studies identified positive associations between parental and preschool children’s physical activity [42,43]. However, Spurrier and colleagues [43] found correlations for parental modelling, particularly maternal participation in physical activity with preschool children’s outdoor playtime. They concluded that during preschool, maternal modelling might have a stronger influence than paternal modelling, which has been found to be more relevant in older children [44]. Nevertheless, our study suggests that the identification with a same-sex model is relevant in preschool children.

Aside from parental correlates, we observed domain-specific relationships between peer and sibling social behaviors and preschool children’s physical activity participation. Concretely, peer support was a relevant predictor of outdoor play, but not for physical activity in or outside of sports clubs, whereas peer modelling was relevant for physical activity in sports clubs and, in boys, also outside of sports clubs. Thus, peers already seem to be important role models and supporters for physical activity in different domains for preschool-aged children, as also shown in previous studies [45,46]. Preschool-aged children should have the chance to mingle with other children in the same or different age groups and to engage in shared physical activities and play.

Moreover, our results demonstrate that sibling modelling was associated with boys’ and girls’ physical activity in sports clubs and with outdoor play in boys only. Preschool children might be motivated to participate in physical activity in sports clubs if they have siblings that do the same. Additionally, younger children may be inspired by older siblings [47], as siblings are potential playmates and thus, facilitate the participation in physical activities together [48,49].

Regarding outdoor play, our study indicates different impacts of parents and siblings for boys and girls, respectively. In early childhood, parents are more protective of girls than boys [50], as fears of strangers or traffic danger are greater for girls than boys [16]. Therefore, parental companionship could be of greater relevance for girls, whereas sibling modelling could be more important for boys [16].

The strength of the current study is its examination of the relationships between the social environment and different domains of physical activity and outdoor play in a nationwide sample of preschool children. We showed that these relationships were different regarding different physical activity domains, and the mechanisms of social influences on children’s physical activity participation seem to differ between physical activities in different domains. Thus, our study contributes to a better understanding of social environmental correlates of physical activity by taking domain-specific physical activity into account instead of focusing on overall physical activity or on MVPA. Furthermore, since our data were drawn from the nationwide MoMo Study conducted in 167 communities in Germany, the results of this study are highly representative.

Nevertheless, some limitations of the study have to be mentioned. First, the data are cross-sectional and do not allow for the analysis of causal relationships. Therefore, we do not know the direction of the relationships found, and it is also possible that the physical activity behavior of the children influenced the social environment instead of the other way around [13,51]. Second, all data was captured from proxy reports and is prone to bias. For example, it is possible that parents misjudged the data. In addition, we did not capture how many fathers and mothers participated in the study, but fathers and mothers may have answered the questionnaire items differently. Furthermore, it is possible that the children did not perceive the same level of support and modelling as gauged by their parents. Thus, we do not know how the questionnaire was answered if there was a disagreement between the child and its parent. Third, only unspecified social support and modelling from parents, peers, and siblings has been considered, and no domain-specific support and modelling data was captured (e.g., parental companionship support for outdoor play or peer support for physical activity in sports clubs). Thus, odds ratios may have been underestimated in our study. Analyzing the relationships of more specific measures of social support and social modelling with the domain-specific physical activity outcomes might lead to even greater odds ratio estimates as found in the current study.

## 5. Conclusions

The present study provides nationwide data from Germany and showed differences of social environmental relationships with physical activity in different domains and in preschool boys and girls. Subsequently, further research should take different domains of physical activity into account to enable the development and successful implementation of programs to promote physical activity. In interventions to promote physical activity in preschool children beside the domain of physical activity, the providers of support and modelling should be targeted. Showing that peer modelling and support were related to a number of domain-specific physical activity measures and interventions including peer groups could be promising in the promotion of physical activity. For the participation in organized sport activities in sport clubs, parents and peers are important providers of support and modelling for both genders and, thus, can be objectives in intervention programs.

## Figures and Tables

**Table 1:** Description of the study sample [*n* (%)].

	Overall (N = 519)	Boys (*n* = 275)	Girls (*n* = 244)
Age			
4 years	188 (36.2)	108 (39.3)	80 (32.8)
5 years	204 (39.3)	96 (34.9)	108 (44.3)
6 years	127 (24.5)	71 (25.8)	56 (23.0)
Socioeconomic status			
low	32 (6.2)	16 (5.8)	16 (6.6)
medium	320 (61.9)	165 (60.2)	155 (63.8)
high	165 (31.9)	93 (33.9)	72 (29.6)
Migration background			
yes	29 (5.6)	11 (4.0)	18 (7.4)
no	490 (94.4)	264 (96.0)	226 (92.6)
Residential area			
rural area	131 (25.2)	71 (25.8)	60 (24.6)
small town	182 (35.1)	99 (36.0)	83 (34.0)
medium-sized town	135 (26.0)	69 (25.1)	66 (27.0)
city	71 (13.7)	36 (13.1)	35 (14.3)
Region in Germany			
former east	169 (32.6)	87 (31.6)	82 (33.6)
former west	350 (67.4)	188 (68.4)	162 (66.4)
Family situation			
living with their biological parents	455 (87.7)	243 (88.4)	212 (86.9)
mother	59 (11.4)	30 (10.9)	29 (11.9)
other	5 (1.0)	2 (0.7)	3 (1.2)

Note: N = total sample size; *n* = group sample size.

**Table 2:** Prevalence of social support, social modelling, and physical activity participation in different domains.

		Descriptive Statistics			Gender Differences	
		Overall	Boys	Girls	T or Chi2	*p*-Value
Peer support	Mean ± SD	2.69 (0.58)	2.66 (0.60)	2.72 (0.55)	−1.14	0.203
Parental emotional support	Mean ± SD	2.96 80.55)	2.93 (0.57)	3.00 (0.52)	−1.45	0.037 *
Parental informational support	Mean ± SD	3.03 (0.56)	3.00 (0.59)	3.07 (0.52)	−1.43	0.316
Parental instrumental support	Mean ± SD	2.93 (0.82)	2.85 (0.83)	3.01 (0.79)	−2.22	0.169
Parental companionship support	Mean ± SD	2.76 (0.58)	2.81 (0.58)	2.71 (0.58)	1.88	0.796
Peer modelling	Mean ± SD	2.75 (0.84)	2.77 (0.81)	2.73 (0.87)	0.58	0.108
Paternal modelling	% active	42.8	43.1	42.4	0.03	0.875
Maternal modelling	% active	50.6	47.6	54.0	2.06	0.151
Sibling modelling	% active	67.8	66.4	69.6	0.48	0.490
Physical activity in sports clubs	% participating	54.1	49.8	59.0	4.41	0.036 *
Physical activity outside of sports clubs	% participating	38.0	35.7	40.4	1.16	0.282
Daily outdoor play	% daily outdoor play	60.7	63.1	58.0	1.38	0.240

Note: * *p* < 0.05, ** *p* < 0.01, *** *p* < 0.001.

**Table 3:** Results of the logistic regression analysis of social support and physical activity in different domains in boys aged 4–6.

	Physical Activity in Sports Clubs				Physical Activity Outside of Sports Clubs				Outdoor Play			
			95% CI				95% CI				95% CI	
	N	Odds Ratio ^1,2^	Lower Bound	Upper Bound	N	Odds Ratio ^1,2^	Lower Bound	Upper Bound	N	Odds Ratio ^1,2^	Lower Bound	Upper Bound
Peer support	253	1.39	0.88	2.21	243	1.31	0.81	2.12	250	2.62 ***	1.58	4.35
Parental emotional support	254	5.33 ***	2.97	9.57	244	2.73 ***	1.56	4.76	251	1.98 **	1.19	3.28
Parental informational support	249	4.56 ***	2.56	8.13	239	2.93 ***	1.68	5.10	246	1.59	0.98	2.58
Parental instrumental support	253	10.61 ***	5.83	19.30	243	1.32	0.91	1.90	250	1.36*	0.95	1.94
Parental companionship support	254	2.06 **	1.28	3.32	244	3.48 **	1.99	6.09	251	1.37	0.87	2.15
Peer modelling	249	3.10 ***	2.03	4.71	239	2.10 ***	1.40	3.15	246	1.20	0.84	1.73
Paternal modelling	249	1.59	0.90	2.80	239	2.17 *	1.20	3.93	246	1.12	0.63	1.99
Maternal modelling	253	1.53	0.88	2.65	243	1.48	0.84	2.61	250	1.42	0.81	2.48
Sibling modelling	255	1.96 *	1.03	3.73	245	1.88	0.92	3.86	252	3.59 ***	1.84	7.03
R^2^ (all predictors and confounders)	0.597 (N = 241)				0.310 (N = 232)				0.242 (N = 238)			

Note: * *p* < 0.05, ** *p* < 0.01, *** *p* < 0.001; ^1^ logit(y) = beta_0_ + beta_1_ *(social support/modelling variable) + [covariates]; ^2^ included covariates are age, socioeconomic status, migration background, residential area (rural, small town, medium-sized town, city), region (east/west), siblings (yes/no), family situation (living at bodily parents/mother/other).

**Table 4:** Results of the logistic regression analysis of social support and physical activity in different domains in girls aged 4–6.

	**Physical Activity in Sports Clubs**				**Physical Activity Outside of Sports Clubs**				**Outdoor Play**			
			**95% CI**				**95% CI**				**95% CI**	
	**N**	**Odds Ratio ^1,2^**	**Lower Bound**	**Upper Bound**	**N**	**Odds Ratio ^1,2^**	**Lower Bound**	**Upper Bound**	**N**	**Odds Ratio ^1,2^**	**Lower Bound**	**Upper Bound**
Peer support	222	1.61	0.93	2.79	215	1.36	0.79	2.36	221	3.08 ***	1.70	5.58
Parental emotional support	222	3.04 ***	1.67	5.54	215	1.28	0.74	2.23	221	2.01 *	1.13	3.56
Parental informational support	218	4.06 ***	2.15	7.64	211	1.22	0.69	2.16	217	2.38 **	1.32	4.31
Parental instrumental support	222	5.51 ***	3.32	9.15	215	1.20	0.83	1.73	221	1.32	0.91	1.91
Parental companionship support	222	1.61	0.95	2.73	215	2.11 **	1.21	3.70	221	2.65 **	1.49	4.70
Peer modelling	216	2.64 ***	1.75	3.98	209	1.04	0.72	1.51	215	1.28	0.89	1.85
Paternal modelling	214	1.24	0.68	2.24	207	0.95	0.52	1.72	213	0.86	0.48	1.57
Maternal modelling	221	1.72	0.96	3.08	214	2.14 *	1.17	3.90	220	1.05	0.58	1.88
Sibling modelling	226	2.43 *	1.23	4.77	219	1.11	0.65	2.19	225	1.07	0.54	2.09
R^2^ (all predictors and confounders)	0.465 (N = 204)				0.163 (N = 197)				0.263 (N = 203)			

Note: * *p* < 0.05, ** *p* < 0.01, *** *p* < 0.001; ^1^ logit(y) = beta_0_ + beta_1_ *(social support/modelling variable) + [covariates]; ^2^ included covariates are age, socioeconomic status, migration background, residential area (rural, small town, medium-sized town, city), region (east/west), siblings (yes/no), family situation (living at bodily parents/mother/other).

## References

[1] Burdette H.L., Whitaker R.C. (2005). Resurrecting free play in young children—Looking beyond fitness and fatness to attention, affiliation, and affect. Arch. Pediatr. Adolesc. Med..

[2] Poitras V.J., Gray C.E., Borghese M.M., Carson V., Chaput J.-P., Janssen I., Katzmarzyk P.T., Pate R.R., Gorber S.C., Kho M.E. (2016). Systematic review of the relationships between objectively measured physical activity and health indicators in school-aged children and youth. Appl. Physiol. Nutr. Metab..

[3] Telama R., Yang X., Leskinen E., Kankaanpää A., Hirvensalo M., Tammelin T., Viikari J.S.A., Raitakari O.T. (2014). Tracking of Physical Activity from Early Childhood through Youth into Adulthood. Med. Sci. Sports Exerc..

[4] Rütten A., Pfeifer K., Banzer W., Ferrari N., Füzéki E., Geidl W., Graf C., Hartung V., Klamroth S., Völker K. (2016). National Recommendations for Physical Activity and Physical Activity Promotion.

[5] Jekauc D., Reimers A.K., Wagner M.O., Woll A. (2012). Prevalence and socio-demographic correlates of the compliance with the physical activity guidelines in children and adolescents in Germany. BMC Public Health.

[6] Eichinger M., Schneider S., De Bock F. (2017). Subjectively and objectively assessed social and physical environmental correlates of preschoolers’ accelerometer-based physical activity. Int. J. Behav. Nutr. Phys. Act..

[7] Hallal P.C., Andersen L.B., Bull F.C., Guthold R., Haskell W., Ekelund U. (2012). Global physical activity levels: Surveillance progress, pitfalls, and prospects. Lancet.

[8] Schmidt S.C.E., Henn A., Albrecht C., Woll A. (2017). Physical Activity of German Children and Adolescents 2003–2012: The MoMo-Study. Int. J. Environ. Res. Public Health.

[9] Farooq M.A., Parkinson K.N., Adamson A.J., Pearce M.S., Reilly J.K., Hughes A.R., Janssen X., Basterfield L., Reilly J.J. (2017). Timing of the decline in physical activity in childhood and adolescence: Gateshead Millennium Cohort Study. Br. J. Sports Med..

[10] Goldfield G.S., Harvey A., Grattan K., Adamo K.B. (2012). Physical Activity Promotion in the Preschool Years: A Critical Period to Intervene. Int. J. Environ. Res. Public Health.

[11] Carver A., Timperio A., Hesketh K., Crawford D. (2010). Are children and adolescents less active if parents restrict their physical activity and active transport due to perceived risk?. Soc. Sci. Med..

[12] Remmers T., Broeren S.M., Renders C.M., HiraSing R.A., Van Grieken A., Raat H. (2014). A longitudinal study of children’s outside play using family environment and perceived physical environment as predictors. Int. J. Behav. Nutr. Phys. Act..

[13] Niermann C.Y., Gerards S.M., Kremers S.P. (2018). Conceptualizing Family Influences on Children’s Energy Balance-Related Behaviors: Levels of Interacting Family Environmental Subsystems (The LIFES Framework). Int. J. Environ. Res. Public Health.

[14] Timperio A., Hume C., Telford A., Cleland V., Salmon J., Crawford D. (2011). A Longitudinal Study of the Family Physical Activity Environment and Physical Activity among Youth. Am. J. Health Promot..

[15] Clements R. (2004). An Investigation of the Status of Outdoor Play. Contemp. Issues Early Child..

[16] Soori H., Bhopal R.S. (2002). Parental permission for children’s independent outdoor activities. Implications for injury prevention. Eur. J. Public Health.

[17] Bandura A. (1986). Social Foundations of thought and Action: A Social Cognitive Theory.

[18] Bussey K., Perry D. (1982). Same-sex imitation: The avoidance of cross-sex models or the acceptance of same-sex models?. Sex Roles.

[19] Ward S.A., Bélanger M.F., Donovan D., Carrier N. (2016). Relationship between eating behaviors and physical activity of preschoolers and their peers: A systematic review. Int. J. Behav. Nutr. Phys. Act..

[20] Uchino B.N. (2004). Social Support and Physical Health: Understanding the Health Consequences of Relationships.

[21] Bauman A.E., Reis R.S., Sallis J.F., Wells J.C., Loos R.J., Martin B.W. (2012). Correlates of physical activity: Why are some people physically active and others not?. Lancet.

[22] Wilk P., Clark A.F., Maltby A., Tucker P., Gilliland J.A. (2018). Exploring the effect of parental influence on children’s physical activity: The mediating role of children’s perceptions of parental support. Prev. Med..

[23] Edwardson C.L., Gorely T. (2010). Parental influences on different types and intensities of physical activity in youth: A systematic review. Psychol. Sport Exerc..

[24] Mendonça G., Cheng L.A., Mélo E.N., Junior J.C.D.F., Júnior J.C.D.F. (2014). Physical activity and social support in adolescents: A systematic review. Health Educ. Res..

[25] Draper C.E., Grobler L., Micklesfield L.K., Norris S.A. (2015). Impact of social norms and social support on diet, physical activity and sedentary behaviour of adolescents: A scoping review. Child Care Health Dev..

[26] Spink K.S., Wilson K.S., Ulvick J. (2012). Social Influence and Adolescent Health-Related Physical Activity in Structured and Unstructured Settings: Role of Channel and Type. Ann. Behav. Med..

[27] Wagner M.O., Bos K., Jekauc D., Karger C., Mewes N., Oberger J., Reimers A.K., Schlenker L., Worth A., Woll A. (2014). Cohort profile: The Motorik-Modul Longitudinal Study: Physical fitness and physical activity as determinants of health development in German children and adolescents. Int. J. Epidemiol..

[28] Kurth B.-M., Kamtsiuris P., Hölling H., Schlaud M., Dölle R., Ellert U., Kahl H., Knopf H., Lange M., Mensink G.B. (2008). The challenge of comprehensively mapping children’s health in a nation-wide health survey: Design of the German KiGGS-Study. BMC Public Health.

[29] Kamtsiuris P., Lange M., Schaffrath Rosario A. (2007). The German Health Interview and Examination Survey for Children and Adolescents (KiGGS): Sample design, response and nonresponse analysis. Bundesgesundheitsbla Gesundheitsforsch Gesundheitsschutz.

[30] Lange M., Butschalowsky H.G., Jentsch F., Kuhnert R., Schaffrath Rosario A., Schlaud M., Kamtsiuris P. (2014). The first KiGGS follow-up (KiGGS Wave 1): Study conduct, sample design, and response. Bundesgesundheitsbla Gesundheitsforsch Gesundheitsschutz.

[31] Schmidt S.C.E., Woll A. (2017). Longitudinal drop-out and weighting against its bias. BMC Med Res. Methodol..

[32] Kurth B.M. (2007). The German Health Interview and Examination Survey for Children and Adolescents (KiGGS): An overview of its planning, implementation and results taking into account aspects of quality management. Bundesgesundheitsbla Gesundheitsforsch Gesundheitsschutz.

[33] Jekauc D., Wagner M.O., Kahlert D., Woll A. (2013). Reliability and Validity of MoMo-Physical-Activity-Questionnaire for Adolescents (MoMo-AFB). Diagnostica.

[34] Schmidt S.C.E., Will N., Henn A., Reimers A.K., Woll A. (2016). Der Motorik-Modul Aktivitätsfragebogen (MoMo-AFB).

[35] WHO (2010). Global Recommondation on Physical Activity for Health.

[36] Reimers A.K., Jekauc D., Mess F., Mewes N., Woll A. (2012). Validity and reliability of a self-report instrument to assess social support and physical environmental correlates of physical activity in adolescents. BMC Public Health.

[37] Schenk L., Ellert U., Neuhauser H. (2007). Children and adolescents in Germany with a migration background. Methodical aspects in the German Health Interview and Examination Survey for Children and Adolescents (KiGGS). Bundesgesundheitsbla Gesundheitsforsch Gesundheitsschutz.

[38] Lampert T., Muters S., Stolzenberg H., Kroll L.E., KiGGS Study Group (2014). Measurement of socioeconomic status in the KiGGS study: First follow-up (KiGGS Wave 1). Bundesgesundheitsbla Gesundheitsforsch Gesundheitsschutz.

[39] Winkler J., Stolzenberg H. (2009). Adjustment of the Social Class Index for Application in the German Health Interview and Examination Survey for Children and Adolescents (KiGGS).

[40] Loprinzi P.D., Trost S.G. (2010). Parental influences on physical activity behavior in preschool children. Prev. Med..

[41] Schoeppe S., Trost S.G. (2015). Maternal and paternal support for physical activity and healthy eating in preschool children: A cross-sectional study. BMC Public Health.

[42] Bringolf-Isler B., Schindler C., Kayser B., Suggs L.S., Probst-Hensch N., The SOPHYA Study Group (2018). Objectively measured physical activity in population-representative parent-child pairs: Parental modelling matters and is context-specific. BMC Public Health.

[43] Spurrier N.J., Magarey A.A., Golley R., Curnow F., Sawyer M.G. (2008). Relationships between the home environment and physical activity and dietary patterns of preschool children: A cross-sectional study. Int. J. Behav. Nutr. Phys. Act..

[44] Ferreira I., Van Der Horst K., Wendel-Vos W., Kremers S., Van Lenthe F.J., Brug J. (2007). Environmental correlates of physical activity in youth? A review and update. Obes. Rev..

[45] Ward S., Bélanger M., Donovan D., Boudreau J., Vatanparast H., Muhajarine N., Leis A., Humbert M.L., Carrier N. (2017). “Monkey see, monkey do”: Peers’ behaviors predict preschoolers’ physical activity and dietary intake in childcare centers. Prev. Med..

[46] Barkley J.E., Salvy S.-J., Sanders G.J., Dey S., Von Carlowitz K.-P., Williamson M.L. (2014). Peer Influence and Physical Activity Behavior in Young Children: An Experimental Study. J. Phys. Act. Health.

[47] Edwards M.J., Jago R., Sebire S.J., Kesten J.M., Pool L., Thompson J.L. (2015). The influence of friends and siblings on the physical activity and screen viewing behaviours of children aged 5–6 years: A qualitative analysis of parent interviews. BMJ Open.

[48] Hesketh K.R., Lakshman R., Van Sluijs E.M.F. (2017). Barriers and facilitators to young children’s physical activity and sedentary behaviour: A systematic review and synthesis of qualitative literature. Obes. Rev..

[49] Irwin J.D., He M., Bouck L.M.S., Tucker P., Pollett G.L. (2005). Preschoolers’ physical activity behaviours: Parents’ perspectives. Can. J. Public Health Revue Can. Sante Publique.

[50] Morrongiello B.A., Dawber T. (1999). Parental influences on toddlers’ injury-risk behaviors: Are sons and daughters socialized differently?. J. Appl. Dev. Psychol..

[51] Ayoub M., Briley D.A., Grotzinger A., Patterson M.W., Engelhardt L.E., Tackett J.L., Harden K.P., Tucker-Drob E.M. (2018). Genetic and Environmental Associations Between Child Personality and Parenting. Soc. Psychol. Personal. Sci..

